# Evaluation of the e–Mental Health Intervention Make It Training From Patients' Perspectives: Qualitative Analysis Within the Reduct Trial

**DOI:** 10.2196/53117

**Published:** 2024-04-09

**Authors:** Julia Barbara Krakowczyk, Femke Truijens, Martin Teufel, Tania Lalgi, Jana Heinen, Caterina Schug, Yesim Erim, Michael Pantförder, Johanna Graf, Alexander Bäuerle

**Affiliations:** 1 Clinic for Psychosomatic Medicine and Psychotherapy LVR-University Hospital Essen University of Duisburg-Essen Essen Germany; 2 Comprehensive Cancer Center University Hospital Essen Essen Germany; 3 Center for Translational Neuro- and Behavioral Sciences University of Duisburg-Essen Essen Germany; 4 Department of Psychology, Educational and Child Studies Erasmus University Rotterdam Rotterdam Netherlands; 5 Department of Psychosomatic Medicine and Psychotherapy University Hospital Tübingen Eberhard Karls University Tübingen Tübingen Germany; 6 Comprehensive Cancer Center University Hospital Tübingen Tübingen Germany; 7 Department of Psychosomatic Medicine and Psychotherapy University Hospital Erlangen Friedrich-Alexander-University Erlangen-Nürnberg Erlangen Germany; 8 Comprehensive Cancer Center University Hospital Erlangen Erlangen Germany; 9 Fraunhofer Institute for Software and Systems Engineering Dortmund Germany

**Keywords:** psycho-oncology, eHealth, digital health, cancer, Reduct trial, oncology

## Abstract

**Background:**

Make It Training is an e–mental health intervention designed for individuals with cancer that aims to reduce psychological distress and improve disease-related coping and quality of life.

**Objective:**

This study evaluated the experienced usefulness and usability of the web-based Make It Training intervention using a qualitative approach.

**Methods:**

In this study, semistructured interviews were conducted with participants at different cancer stages and with different cancer entities. All participants had previously taken part in the Reduct trial, a randomized controlled trial that assessed the efficacy of the Make It Training intervention. The data were coded deductively by 2 independent researchers and analyzed iteratively using thematic codebook analysis.

**Results:**

Analysis of experienced usefulness resulted in 4 themes (developing coping strategies to reduce psychological distress, improvement in quality of life, Make It Training vs traditional psychotherapy, and integration into daily life) with 11 subthemes. Analysis of experienced usability resulted in 3 themes (efficiency and accessibility, user-friendliness, and recommendations to design the Make It Training intervention to be more appealing) with 6 subthemes. Make It Training was evaluated as a user-friendly intervention helpful for developing functional coping strategies to reduce psychological distress and improve quality of life. The consensus regarding Make It Training was that it was described as a daily companion that integrates well into daily life and that it has the potential to be routinely implemented within oncological health care either as a stand-alone intervention or in addition to psychotherapy.

**Conclusions:**

e–Mental health interventions such as Make It Training can target both the prevention of mental health issues and health promotion. Moreover, they offer a cost-efficient and low-threshold option to receive psycho-oncological support.

## Introduction

### Background

Cancer is one of the leading causes of death worldwide, and its prevalence is constantly increasing [1]. Worldwide, 19.3 million new cases of cancer were diagnosed in 2020 [2]. By 2024, a total of 27.5 million new cases of cancer are expected each year [2]. Receiving a cancer diagnosis and undergoing cancer treatment are associated with a high psychological burden [3,4]. Approximately every second individual diagnosed with cancer experiences high psychological distress, and one-third of all individuals across different cancer stages and types meet the criteria for at least one mental health disorder [5-7].

Due to the high psychological burden associated with cancer, a significant number of individuals seek psycho-oncological support [8-10]. Previous research has proven the efficacy of psycho-oncological treatment on different outcomes such as distress, fatigue, depression, anxiety, and quality of life [11-16]. However, receiving proper psycho-oncological support is difficult due to various barriers within the health care system [10,17,18]. These include geographic barriers, the stigma of seeking mental health services, financial constraints, continuity of health care, and the limited availability of mental health professionals [19-21]. Thus, efforts are required to expand access to mental health support for patients with cancer [4,8].

eHealth interventions offer a cost-efficient approach to overcome barriers in psycho-oncological care [16,22,23]. Most of these eHealth interventions consist of (web) applications that are based on psychotherapeutic approaches such as cognitive behavioral therapy (CBT) [24-28]. Existing research has demonstrated the efficacy of psychological eHealth interventions for individuals with cancer on outcomes such as distress, depression, anxiety, fatigue, and quality of life [16,25-27].

Most of the studies evaluating psycho-oncological eHealth interventions have proven their efficacy by adapting a quantitative research approach [16,25-27], wherein statistical analyses are conducted to investigate the pre- and postintervention scores of standardized questionnaires to assess statistically significant differences [29]. Although this approach is considered the gold standard for efficacy research, it does have some limitations [30]. These limitations include missing information on individual experiences, as well as missing in-depth information on the mechanisms behind the change that led to the statistical significance displayed in the data [31]. The inclusion of qualitative research offers an in-depth understanding of these mechanisms [32-34]. Considering research findings from both qualitative and quantitative approaches allows for a more holistic understanding of not only whether an intervention works but also how and why [35,36]. Thus, it offers in-depth knowledge of change mechanisms and the possibility of optimizing existing interventions. Moreover, assessment of eHealth interventions using a mixed methods approach is associated with increased adaptation to patients’ needs and demands compared to solely using quantitative assessments [37-39].

This paper reports qualitative analyses conducted as part of the Reduct trial (German Clinical Trial Register DRKS00025213) [40]. The Reduct trial is a multicenter randomized controlled trial to assess the efficacy of the web-based Make It Training intervention (mindfulness and skill-based distress reduction training in oncology). To date, it is one of the largest efficacy trials in the field of psycho-oncology. Make It Training is a self-guided (web-based) application aimed at reducing distress in individuals with cancer [40,41]. It is based on CBT, acceptance and commitment therapy (ACT), and mindfulness-based stress reduction (MBSR). Over 4 months, individuals are supported by Make It Training through skill training, psychoeducation, interactive exercises, mindfulness, and psychotherapeutic techniques. Make It Training aims to reduce psychological distress, improve disease-related coping, and improve quality of life. It was developed to bridge the gap in the lack of psycho-oncological support in the health care system that currently exists in certain regions. The papers by Bäuerle et al [40] and Heinen et al [41] outline the study and intervention protocols, respectively.

### Study Objectives

Taking on a qualitative stance, this study examined the experienced usefulness and usability of Make It Training from patients’ perspectives. The aim of this study was to obtain a more holistic view and enrich the understanding of individuals’ experiences concerning Make It Training beyond the boundaries of quantitative data [35,36]. When referring to the experience of usefulness, this study took on a psychotherapeutic perspective and referred to the patients’ general evaluation of Make It Training, changes experienced while completing the intervention, attribution of these changes, specific aspects of the intervention that they found particularly useful or hindering, and recommendation to other individuals with cancer. On the basis of the study by Gould and Lewis [42] and the Health IT Usability Evaluation Model [43], the term usability comprises the patients’ experienced user-friendliness, efficiency, accessibility, and practicability of the intervention.

## Methods

### Study Design and Procedure

This study was based on the guidelines of Levitt et al [44] and the COREQ (Consolidated Criteria for Reporting Qualitative Research) guidelines [45]. It consisted of one-on-one semistructured interviews. The interviews were conducted by a trained female interviewer who was experienced with qualitative research. To avoid any potential bias, the interviewer was not part of the core research team of the Reduct trial. There was no previous relationship established between the interviewer and the participants before the study began. Moreover, the participants did not have personal knowledge of the researcher. In total, 33% (2/6) of the participants completed the interviews in person, and 67% (4/6) did so digitally. Apart from the interviewer and the interviewee, there was no other person present during the interviews. All participants were interviewed once. To focus on the dialogue between the interviewee and the interviewer, no field notes were taken during the interviews. No transcripts were returned to the participants for comments or corrections. The COREQ checklist can be found in Multimedia Appendix 1 [45].

### Recruitment

The participants of 1 study center that completed the Make It Training intervention within the Reduct trial [40] between May 2022 and September 2022 were contacted via email and telephone and invited to participate in this study. Purposive sampling (ie, completion of Make It Training) was carried out to obtain information-rich participants as well as in-depth experiences with Make It Training [34,46,47]. Recruitment took place in an early phase of the Reduct trial, so 11 participants were eligible to be contacted in total. Of these 11 participants, 5 (45%) either did not respond or could not participate for personal reasons. The final sample consisted of 6 participants. On the basis of Crouch and McKenzie [48], a small sample size was selected to put emphasis on the relationship between the researcher and the participant, as well as to explore the patients’ lived experiences with Make It Training in depth.

For the inclusion, exclusion, and completion criteria (eg, current cancer diagnosis, command of the German language, internet connection, age of >18 years, and no psychotherapy during the intervention period) of the Reduct trial, we refer to the study protocol by Bäuerle et al [40]. This study was based on the inclusion and exclusion criteria of the Reduct trial.

### Ethical Considerations

This study was approved by the Ethics Committee of the Medical Faculty of the University of Duisburg-Essen (22-10,902-BO). All interviews were conducted on the premises of the university and audiotaped with the interviewees’ consent. The data were pseudonymized. The data protection–compliant audio files and identifying information were stored in a password-protected database. After providing written informed consent, the participants were interviewed. The participants had the option to be interviewed either in person at the clinic or digitally through a data protection–compliant software for clinicians [49]. There was no compensation or any form of reimbursement.

### Semistructured Interview

The interview questions were divided into 9 segments. The first segment focused on explaining the study background and gathering sociodemographic information. In the second to ninth segments, interviewees were asked about the following: general experience with Make It Training, changes that they noticed since completing the intervention, attribution of these changes, content of the intervention that they perceived as particularly helpful or not helpful, content that was perceived as missing, the motivation to participate in the intervention, usability, and recommendation of the intervention to other individuals with cancer.

The interview questions were developed based on the Client Change Interview (CCI) [50] and the Health IT Usability Evaluation Scale (Health ITUES) [51]. The CCI was chosen as it is an established interview within psychotherapy research to assess self-perceived changes and attribution of changes related to psychotherapy [50]. In addition, it helps to identify perceived helpful or unhelpful components of psychotherapeutic interventions [50].

The Health ITUES is a questionnaire used to evaluate the usability of eHealth technologies among people with chronic diseases [52]. It was chosen as it is a validated assessment instrument to evaluate the feasibility and usability of eHealth interventions.

The full version of the semistructured interview is provided in Table S1 in Multimedia Appendix 2 [50,51].

In addition, self-generated questions were included (Textbox 1).

**Self-generated questions of the semistructured interview.**

- How did you perceive the operation and user-friendliness of the Make It Training?
- How did you perceive the additional service in the form of reminder emails and contacts in the event of technical difficulties?

### Data Analysis

The data were analyzed using thematic codebook analysis [53,54]. Thematic analysis was chosen due to its wide application across paradigms [54-56]. An overall deductive approach was chosen because it is an established approach to evaluate user experiences with digital interventions [57]. Moreover, it is helpful in organizing and categorizing meaningful data in conjunction with the existing literature [34,35,54]. The data were coded partly deductively by 2 independent researchers in 2 rounds of analysis. As the research team was interested in the participants’ in-depth lived experiences with Make It Training rather than general thematic cohesion over the sample, a bottom-up inductive analysis was conducted first, which was then captured in the deductive structure in the second round of analysis.

The analyses were conducted iteratively; that is, they were carried out in a cyclical manner to refine and deepen the understanding of the data through the following steps:

1. Each coder open coded the first 2 transcripts, and individual memos were written.
2. The codes were compared and revised through multiple iterative rounds among the research team to obtain different perspectives. Both coders met to compare their findings, particularly regarding the codes; discuss discrepancies to ensure consensus on the application of finalized codes and, if applicable, add new codes; and develop a codebook.
3. Both coders agreed that saturation had been attained in the first 2 open-coded transcripts.
4. The finalized codes were divided into categories and themes [56] and tested on the 4 remaining transcripts.

Chronemics (such as hesitation or silence) were taken into account as nonverbal information in the analysis. Overall, there was a high level of agreement (approximately 70%) between the researchers during the evaluation process, and discrepancies were critically discussed during meetings with the research team to reach a consensus. For publishing purposes, all interview quotes were translated from German into English, and the analysis process was reviewed by the research team. All interviews were transcribed using the f4x transcription software and then analyzed using the MAXQDA computer program (VERBI GmbH) [58]. On the basis of the decision to include a small sample size, the research team defined saturation according to Legard et al [59], meaning that saturation was assessed based on whether there was a consensus among the participants regarding the general evaluation of Make It Training and whether the research team felt that they had reached an understanding of the participants’ lived experiences with Make It Training.

### Quality Control

All researchers involved had a background in clinical psychology, psycho-oncology, psychosomatic medicine, and psychotherapy with different research experiences (full-time professors, assistant professors, postdoctoral researchers, PhD candidates, and graduate students).

On the basis of Creswell and Miller [60], validity guidelines were followed to ensure the validity of this study. These included triangulation by searching for convergence among diverse sources of information (eg, the lens of the researcher and systematic paradigm) to form themes or categories in a study [60]. Finally, validation procedures included seeking assistance through peer debriefing, which was realized by involving an auditor. The auditor was a senior qualitative researcher with extensive experience in clinical psychology and efficacy research but without familiarity with the Reduct trial and the Make It Training intervention. They audited the first round of findings by reading written findings, questioning the researchers on their procedures, and challenging interpretations and thematic structure. Subsequently, the researchers conducted another iterative round of analysis to synthesize and sensitize the data and fine-tune the findings accordingly. To establish credibility, we ensured to provide a thick and rich description of the setting, participants, and themes of the qualitative study [61].

## Results

### Overview

A total of 6 (mean 34 min, SD 7 min 56 s; range 20-45 min) one-on-one interviews were conducted. The demographic characteristics of the participants are presented in Table 1.

**Table 1:** Sociodemographic and diagnosis-related characteristics of the participants (N=6).

Characteristics			Participants, n (%)
**Gender^a^**			
	Identified as a woman	4 (67)	
	Identified as a man	2 (33)	
**Age range (y)^a^**			
	49-56	4 (67)	
	57-66	2 (33)	
**Cancer type^b^**			
	Breast cancer	1 (17)	
	Lymphatic; blood-forming tissue	1 (17)	
	Skin cancer	2 (33)	
	Colon cancer	1 (17)	
	Musculoskeletal tumors	1 (17)	
**Year of initial cancer diagnosis^b^**			
	2010	1 (17)	
	2018	1 (17)	
	2019	1 (17)	
	2020	2 (33)	
	2022	1 (17)	
**Recurrence^b^**			
	Yes	5 (83)	
	No	1 (17)	
**Metastasis^b^**			
	Yes	3 (50)	
	No	3 (50)	

^a^Sociodemographic characteristic.

^b^Medical characteristic and etiopathology.

### Theme Classification

#### Overview

**Figure 1:**
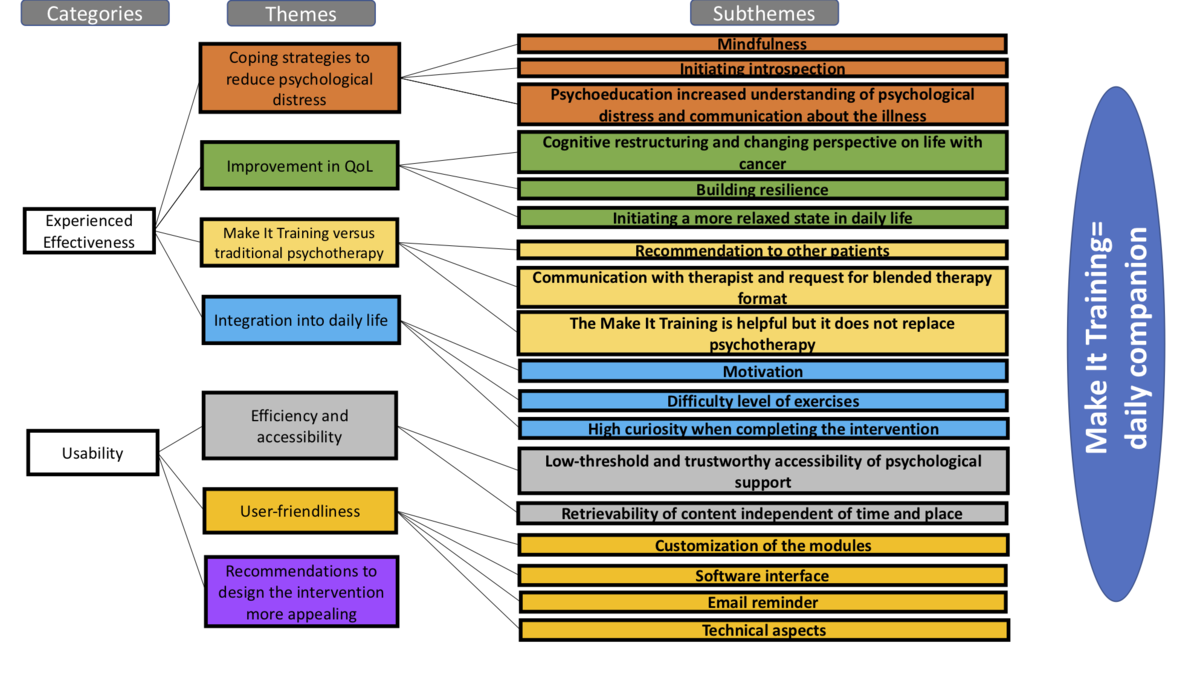
Themes and subthemes from the codebook analysis. Graphic display of the overarching categories, themes, and subthemes that emerged during the data analysis process. The term daily companion refers to the term that was commonly used by the participants to describe the Make It Training intervention. QoL: quality of life.

The previously selected categories were divided into 7 themes that were used to focus the qualitative analyses. The themes were used deductively to select excerpts in the interviews that appeared relevant to these themes. Within the selections per theme, excerpts were coded using line-by-line coding and grouped to form information-rich subthemes. All themes and subthemes are reported in the following sections using representative quotes. Further information on the theme classification can be found in Figure 1, whereas Table S2 in Multimedia Appendix 2 summarizes all representative quotes.

The consensus regarding the Make It Training intervention was that it was described as a “daily companion” that integrates well into daily life and that it has the potential to be routinely implemented within oncological health care either as an intervention itself or in addition to psychotherapy (Table S2 in Multimedia Appendix 2, quote 1).

#### Category 1: Experienced Usefulness

##### Theme 1: Developing Coping Strategies to Reduce Psychological Distress

###### Overview Theme 1

This theme is centered on the development of functional coping strategies that participants described as a change related to Make It Training. All participants reported that Make It Training helped them develop a repertoire of coping strategies, which was helpful in reducing psychological distress.

For example, the improvement in emotion regulation was described as such a strategy (Table S2 in Multimedia Appendix 2, quote 2). Another commonly described coping strategy was redefining the relationship with cancer (Table S2 in Multimedia Appendix 2, quote 3).

###### Subtheme 1.1: Mindfulness Exercises

The increased practice of mindful behavior stood out as a described coping strategy, and it was attributed to the mindfulness exercises provided in Make It Training. The participants strongly embraced the variety of mindfulness exercises provided in the intervention. Interviewee 6 would have preferred even more exercises within Make It Training (Table S2 in Multimedia Appendix 2, quote 4).

The mindful breathing exercises were most commonly described as helpful. They were perceived as a new coping skill that could be integrated into daily life for stress management and tension reduction. One of the participants also positively noted the long-term advantages of breathing exercises (Table S2 in Multimedia Appendix 2, quote 5). This statement illustrates the advantages of mindful breathing exercises as part of the coping repertoire. Moreover, it demonstrates the practical application of the techniques in daily life as well as the interviewees’ subjective perception of improvement.

###### Subtheme 1.2: Initiating Introspection

Most participants reported that Make It Training helped initiate introspection, which was described as supportive in dealing with difficult situations. It was further described as developing the skill to observe and interpret one’s own thinking patterns, emotions, and behavior and not just be overwhelmed by them (Table S2 in Multimedia Appendix 2, quotes 6 and 7). Moreover, being able to observe one’s inner world (ie, introspection) can help shift attention to positive aspects in difficult phases (Table S2 in Multimedia Appendix 2, quote 8).

###### Subtheme 1.3: Psychoeducation Increased Understanding of Psychological Distress Associated With Cancer and Communication About the Illness

Many participants experienced the psychoeducational components within the intervention as helpful because they led to a better understanding of cancer and its associated psychological distress and somatic restrictions. The participants reported that they were able to learn not only about personal circumstances but also how to communicate better and more effectively approach family members. In this regard, the expert videos provided, where health care professionals reported on each topic, were perceived as useful (Table S2 in Multimedia Appendix 2, quote 9).

##### Theme 2: Improvement in Quality of Life

###### Overview Theme 2

All participants reported that Make It Training helped increase their quality of life. This was described as redefining perspectives on life circumstances and cancer. Moreover, health-related behavior change, increase in resilience, and enhanced practice of mindful behavior were described as positively contributing to quality of life (Table S2 in Multimedia Appendix 2, quote 10).

###### Subtheme 2.1: Cognitive Restructuring and Changing Perspective on Life With Cancer

Participants reported that Make It Training helped modulate existing thinking patterns. This was commonly described as changing perspectives on life with cancer, as well as on the cancer diagnosis itself (Table S2 in Multimedia Appendix 2, quote 11). Another participant described a redefined relationship with pain (Table S2 in Multimedia Appendix 2, quote 12).

###### Subtheme 2.2: Building Resilience

Participants reported that Make It Training helped them become more resilient, which was described as developing the ability to better deal with unpleasant situations such as chemotherapy (Table S2 in Multimedia Appendix 2, quote 13).

###### Subtheme 2.3: Initiating a More Relaxed State in Daily Life

The participants described that the intervention was helpful to experience a more relaxed state in daily life, which positively contributed to their quality of life (Table S2 in Multimedia Appendix 2, quotes 14 and 15).

##### Theme 3: Make It Training Versus Traditional Psychotherapy

###### Overview Theme 3

While evaluating the Make It Training intervention, some participants drew a comparison between Make It Training and traditional psychotherapy. In total, 33% (2/6) of the participants had previous psychotherapeutic experience. Even though Make It Training was perceived as a helpful and easily accessible format to receive psycho-oncological support, 83% (5/6) of the patients reported that it did not replace traditional psychotherapy (Table S2 in Multimedia Appendix 2, quote 16). In contrast, one participant reported preferring Make It Training to traditional face-to-face psychotherapy (Table S2 in Multimedia Appendix 2, quote 17).

###### Subtheme 3.1: Recommendation to Other Patients

All participants had been diagnosed with different cancer entities and stages (Table 1). Overall, all reported recommending Make It Training to others as they were convinced that other individuals with cancer could benefit from the intervention as well. Some of them suggested that a psycho-oncological eHealth intervention such as Make It Training should be offered as a routine intervention within oncological health care.

Multiple participants argued that particularly individuals with a first-time cancer diagnosis would substantially benefit from the intervention. One participant hypothesized that providing individuals with a first-time diagnosis of cancer with an eHealth application such as Make It Training would help them process and better deal with the cancer diagnosis (Table S2 in Multimedia Appendix 2, quotes 18 and 19).

###### Subtheme 3.2: Communication With Therapist and Request for Blended Therapy Format

Make It Training is a purely self-guided eHealth intervention. Some participants wished for more communication with a therapist. In this context, they stressed the importance of a patient-therapist interaction. Some participants reported that Make It Training might be even more beneficial with additional therapist guidance. In this regard, additional therapist consultations via phone or email were suggested. Moreover, participants reported that these options would offer the opportunity to better voice challenges, misunderstandings, and questions. A total of 50% (3/6) of the participants expressed a preference for a blended format (ie, a combination of Make It Training with traditional face-to-face psychotherapy; Table S2 in Multimedia Appendix 2, quote 20).

##### Theme 4: Integration Into Daily Life

###### Overview Theme 4

The intervention was described as a “daily companion” (interviewee 4) or “a wonderful companion for everyday life” (interviewee 2) that could help a lot of individuals with cancer. Make It Training provided participants with a variety of psychoeducational information, psychotherapeutic exercises, and skill training that were perceived as suitable for integration into daily life. All participants reported that they had incorporated the received information or skills that they found valuable and implementable (see Table S2 in Multimedia Appendix 2, quotes 21 and 22, for examples of how participants integrated the skills into their daily lives).

###### Subtheme 4.1: Motivation

In the initial phase, all participants reported being motivated to complete the intervention. However, there were divided opinions regarding motivation after that initial phase. Some experienced Make It Training to be action activating because “it was a meaningful engagement with the disease” (interviewee 2). For others, the motivation gradually declined.

One participant brought up an analogy from sports to describe their motivation. They addressed the fact that, over time, they lacked the motivation to continue through Make It Training. However, the reminder emails helped keep the participant motivated (Table S2 in Multimedia Appendix 2, quote 23). In contrast, there were participants who did not need an external motivator (Table S2 in Multimedia Appendix 2, quotes 24-26).

###### Subtheme 4.2: Difficulty Level of Yoga Exercises

Make It Training comprised physical exercises in the form of yoga. There were mixed opinions on the difficulty level of these exercises as some participants perceived them as physically exhausting, whereas others did not. An older participant reported that some physical exercises were too straining due to restrictions caused by a lack of mobility because of the cancer (Table S2 in Multimedia Appendix 2, quote 27).

###### Subtheme 4.3: High Curiosity When Completing the Make It Training Intervention

Curiosity was high among all participants to see “what’s new there?” (interviewee 1) when a new module was unlocked. Curiosity was described as high because one had to wait a week to unlock a new module, which was perceived as exciting (Table S2 in Multimedia Appendix 2, quotes 28-30). Overall, participants seemed to support the format in which content is unlocked incrementally as it generates curiosity.

#### Category 2: Usability

##### Theme 5: Efficiency and Accessibility of the Make It Training Intervention

###### Overview Theme 5

The digital setup allowed all participants to work through the modules independent of time and place. Because of that, Make It Training was perceived as an efficient and easily accessible format to receive psycho-oncological support (Table S2 in Multimedia Appendix 2, quote 31).

###### Subtheme 5.1: Low-Threshold and Trustworthy Accessibility of Psychological Support

The content provided during the intervention was perceived as professional and trustworthy. It was reported that having access to Make It Training was not associated with barriers that were previously experienced by some participants when seeking psychotherapy. This was perceived as very positive (Table S2 in Multimedia Appendix 2, quotes 32-34).

###### Subtheme 5.2: Retrievability of Content Independent of Time and Place

All participants positively outlined the retrievability of the content. This refers to the possibility to flexibly retrieve the contents of Make It Training independent of time and place. When a module is activated, the participants can choose when and for how long they want to work on it, as well as on what parts. This was perceived as useful as it offers the flexibility to work on the modules independently of physicians’ appointments, operations, or other medical examinations. Thus, Make It Training was considered “really timely-ideal” (interviewee 4; Table S2 in Multimedia Appendix 2, quote 35).

Participants also reported that the retrievability of the content helped them assess whether a skill that was learned could actually be internalized as well, which was perceived as a benefit (Table S2 in Multimedia Appendix 2, quotes 36 and 37).

##### Theme 6: User-Friendliness

###### Overview Theme 6

There were mixed opinions regarding the user-friendliness of Make It Training. Overall, participants considered the application user-friendly. One of the most common reasons why the intervention was described as user-friendly was that it was perceived as not requiring much guidance when using it (Table S2 in Multimedia Appendix 2, quote 38).

One participant criticized the user-friendliness of Make It Training because they perceived the software interface as confusing (Table S2 in Multimedia Appendix 2, quote 39).

###### Subtheme 6.1: Customization of the Modules

Make It Training follows a certain chronology in the order of the modules, which is not customizable. This was experienced by most participants as very limiting, and they would have liked to be able to work through the modules in their own order (Table S2 in Multimedia Appendix 2, quote 40).

###### Subtheme 6.2: Software Interface

There were mixed opinions regarding the software interface of Make It Training. Some participants perceived the layout of Make It Training as clear and stimulating. In contrast, others pointed out the unclear and childish presentation of the modules. One participant also came up with an analogy to a “kids board game” (interviewee 5). In general, the rather playful approach was appreciated (Table S2 in Multimedia Appendix 2, quotes 41 and 42).

###### Subtheme 6.3: Email Reminder to Increase Adherence

There were mixed opinions regarding the reminder emails that all participants received throughout the intervention. Most perceived them as a helpful addition that encouraged them; however, some of the participants perceived them as a bother (Table S2 in Multimedia Appendix 2, quote 43).

###### Subtheme 6.4: Technical Aspects

Most of the participants did not report any significant technical difficulties or perceived deficiencies. Common technical issues included internet connection or low-resolution quality of the videos.

##### Theme 7: Recommendations to Design the Make It Training Intervention to Be More Appealing

The participants gave feedback on how to design the Make It Training intervention to be more appealing. One module that focused on the family members of individuals with cancer was regarded by 33% (2/6) of the participants as lacking sensitivity. They reported that working through this module seemed inappropriate and upsetting for those without family members (Table S2 in Multimedia Appendix 2, quotes 44 and 45).

As another recommendation, some participants expressed the need to adapt the modules to the stage of cancer and the current treatment phase (Table S2 in Multimedia Appendix 2, quote 46).

Regarding usability, participants reported minor technical issues or design shortcomings that affected their navigation of and interaction with the program (eg, struggle to remember their position or progress within the program and challenges in finding the right areas to click or interact with). Clearer indicators or visual cues to help users track their progress and easily identify their current location within the program’s content or structure were suggested (Table S2 in Multimedia Appendix 2, quotes 47-49).

## Discussion

### Principal Findings

This study examined the experienced usefulness and usability of Make It Training from patients’ perspectives using a qualitative approach, which was accomplished through thematic analysis of interviews conducted with individuals with cancer at different stages of severity. Analysis of their experience of the usefulness of Make It Training resulted in 4 themes (developing coping strategies to reduce psychological distress, improvement in quality of life, Make It Training vs traditional psychotherapy, and integration into daily life) with 11 subthemes. Analysis of their experienced usability resulted in 3 themes (efficiency and accessibility, user-friendliness, and recommendations to design the Make It Training intervention to be more appealing) with 6 subthemes. All participants positively evaluated Make It Training. Moreover, all participants reported that they experienced positive changes while completing the Make It Training intervention and attributed these changes to the intervention itself. The overall usability of Make It Training was experienced as positive as well, although the experiences showed variation due to personal preferences. Overall, the results of this study point to a high satisfaction with Make It Training.

The themes that were discussed as perceived changes during the Make It Training intervention are consistent with its overall goal, which is to support individuals with cancer with disease-related coping, improvement in quality of life, and reduction in psychological distress [40,41]. Moreover, the aforementioned results are in line with those of the study by Ringwald et al [62], who assessed the acceptance of and satisfaction with a previous version of the Make It Training intervention in a pilot study. In this study, the acceptance and satisfaction rates of Make It Training were high, and 87% of the participants reported that they would recommend the intervention to other individuals with cancer [62]. Overall, the results from both the study by Ringwald et al [62] and our study point to a high acceptance of and satisfaction with Make It Training. Because of their satisfaction with Make It Training, the participants stated that it should be implemented as a routine intervention within health care. Previous research has shown that there is a relationship between acceptance of eHealth interventions and their actual use [63-67]. Acceptance is also an important factor for adherence [68]. Thus, given the acceptance of and satisfaction with Make It Training, it might have potential as an eHealth intervention to be routinely implemented in oncological health care as a medical device. In Germany, for example, there is a more recent regulation that eHealth interventions can be prescribed by health care professionals.

The Make It Training was described as a low-threshold and efficient format to receive psycho-oncological support. This was perceived as extraordinarily helpful as some participants had previously experienced difficulties with receiving proper psycho-oncological support, which is known to be a common problem in certain regions [10,17,18]. In this regard, the retrieval of content independent of time and place was described as being helpful with internalizing learned skills and accessing psychological support quickly when needed. These results further support the implementation of eHealth interventions such as Make It Training as an integral part of oncological health care. Digital interventions, if they are accepted among users, can overcome barriers associated with receiving psychological support, thereby improving mental health care and aftercare in oncology [19-21]. As individuals with cancer show elevated levels of distress both during and after cancer treatment, access to (digital) mental health care within this field is of great importance for both prevention and health promotion [4,8].

Despite mixed opinions regarding the software interface, Make It Training was generally rated as user-friendly. The participants most commonly argued for the usability of Make It Training by discussing that high technological literacy was not a requirement for completing the intervention. This finding is consistent with those of previous research showing a link between the use and acceptance of eHealth interventions and users’ technological literacy [23,66,67]. Even though eHealth interventions have the potential to improve health care and aftercare, their implementation often fails because patients face barriers when wanting to make use of these interventions [23,66,67,69]. These barriers include low technological literacy, limitations in technological access, limitations in usability, and limited education in digital advice [69-72]. In addition, there are demographic barriers based on differences in age, socioeconomic status, educational level, language, and culture. Overall, existing barriers to receiving digital interventions due to demographic or structural differences can foster insensitivity within health care [72-74]. Certain individuals with cancer are at risk of being excluded from digital interventions because this population tends to have a higher median age (>60 y) [75], whereas the disease affects individuals with all kinds of demographic characteristics (ie, different cultural backgrounds, socioeconomic statuses, and educational levels). In addition, individuals commonly experience cognitive and physical restrictions during cancer treatment [76]. Thus, for more inclusive health care for individuals with cancer, eHealth interventions need to be designed as barrier free as possible (ie, they should depend less on the user’s technological literacy as well as on other potentially exclusive factors).

Make It Training was compared by the participants to traditional face-to-face therapy even though it was not a specific topic in the interviews. In this regard, Make It Training was described as a helpful intervention, although it was noted that it could not replace traditional psychotherapy. The participants reported the missing therapist interaction as the main reason. In this regard, there was a desire for more therapist interaction within the Make It Training. In addition, a blended therapy format (ie, a combination of the Make It Training with additional face-to-face psychotherapy) was described as the “ideal” format to receive psycho-oncological support. This is in line with previous research supporting the adaptation of blended therapy approaches in psycho-oncology as well [77]. Efficacy research shows that purely self-guided eHealth interventions are associated with smaller effect sizes with a lower completion rate compared to blended therapy interventions, which can be attributed to the missing therapist interaction [78,79]. The results of this study, along with existing research, indicate that it is highly important to adapt eHealth interventions to the patients’ needs [80]. Thus, it is suggested to put emphasis on therapist interaction (ie, blended format) in psycho-oncological eHealth interventions.

In this study, a qualitative approach was chosen as we believe that the inclusion of qualitative analyses within efficacy research (ie, the Reduct trial; Bäuerle et al [40]) provides more scientifically sound and transportable results. In this regard, it is important to look beyond surface or aggregate-level evidence to allow for inter- and intrapersonal nuances [81]. These are often missed in efficacy research but are rather important for a holistic understanding of usefulness in clinical practice [81]. Including qualitative research allows for an investigation of these inter- and intrapersonal nuances as well as for scrutiny of the level of experience, which is an important aspect when evaluating health care interventions such as the Make It Training. Another important strength of this study is the heterogeneity of the sample (ie, all participants were diagnosed with different cancer types and stages), which positively contributed to the generalizability of the evaluation of the Make It Training. In addition, this study provided the research team with information-rich descriptions of the participants’ lived experiences regarding the Make It Training. It was also possible to obtain in-depth feedback on how to design the Make It Training intervention to be more appealing from a patient’s perspective. Practical implications derived from this study are, from patients’ perspectives, the potential of psycho-oncological eHealth interventions such as the Make It Training to improve oncological health care by offering a low-threshold option that provides psychological support independent of time and place and does not interfere with the already time-consuming oncological treatment. However, for routine implementation, they need to be adapted to the patients’ needs and designed to be barrier free and should not require high technological literacy to interact with them. Moreover, even though eHealth interventions do offer efficient psycho-oncological support, they do not replace traditional psychotherapy, and it is suggested to use them as a first-step psychological support in a stepped-care health care approach.

### Limitations and Recommendations for Future Research

This study has some limitations. Even though a qualitative approach offers valuable insights into participants’ in-depth experiences, there are limitations regarding qualitative research itself, particularly concerning its generalizability and objectivity [82]. In this study, the decision to use a small sample size might have had a negative impact on the generalizability of the results even though the research team made efforts to select a highly heterogeneous sample. Moreover, a small sample size leads to a smaller data corpus, which can negatively impact the achievement of full thematic saturation. Other limitations include the use of a deductive analysis approach [54] and the risk of selection bias. Moreover, most of the research team members have a background primarily in quantitative methodology. Even though attempts were made to reduce this potential bias by actively involving an expert in qualitative research, this should still be considered a limitation. On the basis of the results of this study, it is suggested that future research put more emphasis on the barrier-free design of interventions and include patients’ perspectives when designing and evaluating eHealth interventions. Moreover, it is suggested that future research investigate blended therapy approaches (ie, a combination of digital psycho-oncological interventions and face-to-face psychotherapy) as this format seems to be appealing for individuals with cancer.

### Conclusions

The Make It Training was evaluated as a user-friendly intervention that is helpful for developing functional coping strategies to reduce psychological distress and improve quality of life among individuals with cancer. It has the potential to be implemented as a routine eHealth intervention in oncological health care. Overall, the results of this study, along with the existing literature, support the paradigm shift of including digital mental health care in the treatment of somatic and mental health disorders. e–Mental health interventions such as Make It Training can target both prevention of mental health issues and health promotion and offer a cost-efficient and low-threshold option to receive psycho-oncological support. Moreover, they allow for the retrieval of mental health support content independent of time and place. However, for psycho-oncological eHealth interventions to be actually used, they need to be designed to be barrier free and adapted to the users’ needs.

## Appendix

Multimedia Appendix 1 COREQ (Consolidated Criteria for Reporting Qualitative Research) checklist.

Multimedia Appendix 2 Semistructured interview questions and quotes.

## Abbreviations

ACT: acceptance and commitment therapy
CBT: cognitive behavioral therapy
CCI: Client Change Interview
COREQ: Consolidated Criteria for Reporting Qualitative Research
Health ITUES: Health IT Usability Evaluation Scale
MBSR: mindfulness-based stress reduction

## Data Availability

The data sets generated during and analyzed during this study are available from the corresponding author on reasonable request.
