# Supplementary material for: Evaluation of the e–Mental Health Intervention Make It Training From Patients' Perspectives: Qualitative Analysis Within the Reduct Trial
Source: JMIR Cancer. 2024 Apr 9;10:e53117. doi: 10.2196/53117 (PMC11040444; doi:10.2196/53117)
Supplement: Multimedia Appendix 2 [file cancer_v10i1e53117_app2.docx]

## Multimedia Appendix 2

Table S1. Semistructured interview.

| Segments ^a^ | Questions | Source ^b^ |
| --- | --- | --- |
|  |  |  |
| General experience of the intervention | 1. How did you experience the Make It Training? | based on Client Change Interview [50] |
| Changes | 1. Are there any changes in you that you have noticed since the Training? | based on Client Change Interview [50] |
| Attribution | 1. What do you think brought about these changes? | based on Client Change Interview [50] |
| Helpful aspects/ content | 1. Were there aspects/content of Make It Training that you found helpful? 2. Which aspect/content was the most helpful? 3. What about it did you find most helpful? 4. What do you think it was because of? | based on Client Change Interview [50] |
| Obstructive aspects/content | 1. Were there aspects/contents that were not helpful, hindering, negative or disappointing for you? 2. What caused these aspects to be unhelpful? 3. What would you have liked instead? | based on Client Change Interview [50] |
| Missing aspects/ content | 1. In your opinion, what content in the Make It Training fell short or was missing? | based on Client Change Interview [50] |
| Motivation | 1. How easy was it for you to get motivated to work with the Make It Training? | based on Client Change Interview [50] |
| Usability | 1. How did you feel about the additional services in the form of reminder emails and contact persons in case of technical difficulties? | based on self-generated questions |
| Recommendation | 1. Would you have any suggestions or recommendations to make the Make It Training more appealing? | based on Client Change Interview [50] |
|  | 1. To what extent do you think the Make It Training would improve the quality of life for people with cancer. | based on Health-Information Technology Usability Survey (Health ITUES) [51] |
|  | 1. If a friend needed similar help, would you recommend our Make It Training to him or her? | based on Client Change Interview [50] |

^a^Semi-structured interview that was conducted in the present study. The interview is based on Elliott et al. [50] and Yen et al. [⁠[51].

Table S2. Tabular listing of all verbatim quotes of the participants (N=6) structured according to the themes and subthemes from codebook analysis.

| Theme classification | | Quotes |
| --- | --- | --- |
| Overall feedback | | - Quote 1: “Yes, I found the overall package very, very interesting for me. I also felt picked up, so from all the information I got.” [Interviewee 3] |
| **Category 1: experienced usefulness** | | |
|  | Theme 1: developing coping strategies to reduce psychological distress | - Quote 2: “[...] it was also a recurrence in my case now. The first time, I was able to cope with everything. But now, of course, the fear has become stronger that something will come back and so on, but now I can deal with it better thanks to the Make It Training.” [Interviewee 2] - Quote 3: “And that is, of course, why this Make It Training has helped with the major problem in dealing with this major problem und has the perception of the disease less, or even the threat of the disease reduced, I would say even dramatically reduced.” [Interviewee 5] |
|  | Subtheme 1.1: mindfulness exercises | - Quote 4: “I would have liked overall actually more these, these these mindfulness exercises. In fact, two in each module would have been preferable [...] and more the the experts who tell you something about it.” [Interviewee 6] - Quote 5: “What was particularly helpful for me were these breathing exercises. So I really integrated them into my everyday life. And I had a situation yesterday where I was incredibly excited and I couldn’t control it anymore. That was so bad. I went to an appointment even though it wasn’t anything dramatic. Not at all. But I was so excited. And then I did breathing exercises along the way to really say it’s nothing, Come down now and it’s all good. And I really did that exactly as described in this Make It Training with holding the breath, breathing out slowly, breathing in again, feeling the breath like that. And I really felt like that for me, it’s getting better.” [Interviewee 4] |
|  | Subtheme 1.2: initiating introspection | - Quote 6: “[...] To stand on another point of view, to judge worries. So just when. If the worries get out of hand and you somehow panic or something, that you then try to get out of yourself and observe yourself from the outside, so to speak, and see that it’s actually useless and that you’d better stay calm.” [Interviewee 2] - Quote 7: “Between chemo therapies. To clear my head a bit and still deal with it, because suppressing it is not the right method. And that was quite good.” [Interviewee 2] - Quote 8: “And I think the study helps a little bit to pause this time and look and say, man, it’s beautiful. Nevertheless, it is beautiful. And I think that’s quite important.” [Interviewee 4] |
|  | Subtheme 1.3: psychoeducation increased understanding of psychological distress associated with cancer and communication about the illness | - Quote 9: “It was extremely informative for me, very factually oriented, made by trustworthy specialists, so that I could also absorb all the things that were said there, how should I put it, uncritically anyway, but trustworthily.” [Interviewee 5] |
|  | Theme 2: improvement in quality of life | - Quote 10: “An extreme improvement in the quality of life [...] One of the...actually the most helpful thing that I’ve come across now. I think. So, I, I this is such an experience in life...is this Make It Training there is an experience in life, where I think, that had to make actually much earlier times. So that I profit and not only illness-related simply from it...I also profit extremely from it in my life.” [Interviewee 5] |
|  | Subtheme 2.1: cognitive restructuring and changing perspective on life with cancer | - Quote 11: “As I said, it’s this self-reflection. Of course, when you’re in pain and you’re facing another operation with an uncertain outcome, that’s difficult, but for me it’s also very important to always say. Yes, but life still goes on. And even with this diagnosis, it’s not over yet.” [Interviewee 4] - Quote 12: “Yes, I saw some things a little differently. For example, above all the point about pain. At that time I had insane pain and then somehow this sentence of because pain can also be positive and that has already given me a transformation in thinking that I, that I saw the pain also in relation to the cell renewal injection for example then positive. But actually something positive happened in my body.” [Interviewee 1] |
|  | Subtheme 2.2: building resilience | - Quote 13: “I have to say, for me it was just right because that was just in my low situation and with all the pushing back and forth or and do here. Would have been completely lost on me, because somewhere through all the treatments up here was completely not okay. So I’m very grateful for that.” [Interviewee 1] |
|  | Subtheme 2.3: initiating a more relaxed state in daily life | - Quote 14: “Yes, it actually helped me in general to find better relaxation in many things like that in the first place. [...] As I said, the breathing exercises were quite great.” [Interviewee 2] - Quote 15: “[...] At some point you’re so over the hill and then it drops again. And actually primitive in retrospect. But I don’t think I would have thought of it that way myself. And when you see it like that, then you realize Yes, that’s right, it’s been like that in the past and it will be like that the next time. And of course that makes you feel much less anxious, because the situation can’t continue to increase indefinitely. You know, you have to endure it for a certain time and that will eventually get better again.” [Interviewee 5] |
|  | Theme 3: Make It Training vs traditional psychotherapy | - Quote 16: “What I have to say, though, is that a face-to-face conversation cannot be replaced by the Make It Training. So this exchange between therapist and patient, that is essential.” [Interviewee 4] - Quote 17: “So compared to the actual personal therapy. So I have to say that the Make It Training was significantly more beneficial to me.” [Interviewee 5] |
|  | Subtheme 3.1: recommendation to other patients | - Quote 18: “I also took that to my fellow patients where I knew they had no experience at all. People, get into the study, try to get into it. It will help you. For someone who really has absolutely no idea about it, I think it’s very helpful.” [Interviewee 1] - Quote 19: “I can imagine that this helps a lot of first-time sufferers incredibly, because you’re just so empty at that moment.” [Interviewee 6] |
|  | Subtheme 3.2: communication with therapist and request for blended therapy format | - Quote 20: “[...] it would have been very nice to have the opportunity to ask questions directly. [...] It’s not so bad that you have a question that you could type in and then send it off, for example. And then at the end of the session or maybe at the next session, that you would get an answer or, if possible to the email address somehow get an answer. That would be very nice, because sometimes any questions arise then.” [Interviewee 4] |
|  | Theme 4: integration into daily life | - Quote 21: “So once a week to really get something like that, to get your unit, to work through it, then to review it again like that. [...] Practically in every module there was something where I also said that would be nice or I would like to try that again.” [Interviewee 4] - Quote 22: “Or these breathing exercises, which help me tremendously. And that I can practically integrate them into my everyday life. Or if necessary, as I said, that I was so, so excited, I can use them again as an aid.” [Interviewee 4] |
|  | Subtheme 4.1: motivation | - Quote 23: “And at first always euphoric and interested. And that leaves like everything else in life, whether that’s an athletic course or no matter what it is, everything wears off a bit at some point. Yes, if you’re not completely disciplined.” [Interviewee 3] - Quote 24: “So once a week to really get something like that, to get your unit, to work through it, then to review it again like that, because always...Practically in every module there was something where I said that would be nice or I would like to try that again.” [Interviewee 4] - Quote 25: “I had the feeling right from the start that this would be extremely helpful for me. And that’s why that was motivation enough to improve my situation.” [Interviewee 5] - Quote 26: “Yeah, it motivated me. Now get off your butt and do it.” [Interviewee 1] |
|  | Subtheme 4.2: difficulty level of yoga exercises | - Quote 27: “Yeah, the difficulty level. This squatting on the floor and stuff and then also getting up off the floor afterwards.” [Interviewee 2] |
|  | Subtheme 4.3: high curiosity when completing the Make It Training intervention | - Quote 28: “Well, I was always looking forward to it, when a new module was released, where I could deal with it.” [Interviewee 2] - Quote 29: “But I enjoyed doing it. I have to say, I always looked forward to it and then I always looked. Aha, next module is already on again, I can work on it now. And yes, I always enjoyed doing that. I’ve actually always waited for it.” [Interviewee 4] - Quote 30: “The nice thing is that you could never leaf through it like a book. You always had to wait week by week, because what comes next. Yes, yes, that is, I think, quite good. It makes you curious to always be on the ball.” [Interviewee 3] |
| **Category 2: usability** | | |
|  | Theme 5: efficiency and accessibility of the Make It Training intervention | - Quote 31: “In purely practical terms, it is also relatively difficult to find a therapist quickly. At least that’s my experience. [...] But you can actually do a Make It Training here immediately after five minutes, you have immediate access.” [Interviewee 5] |
|  | Subtheme 5.1: low-threshold and trustworthy accessibility of psychological support | - Quote 32: “At the end of the day, it was kind of like I’m going to see a psychologist when I clicked on the module. Kind of like that a little bit.” [Interviewee 3] - Quote 33: “And I thought that was really great, because before you normally start talking to a specialist in a field of medicine, or you can, you need a lot of hurdles. And they’re not here at all. You have immediately. You have a lecture by an expert, who stands there for you and formulates this to you in a general way and says pay attention to it, because then there was something with partners.” [Interviewee 6] - Quote 34: “It was extremely informative for me, very factually oriented, made by trustworthy specialists, so that I could also absorb all the things that were said there, how should I put it, uncritically anyway, but trustworthily.” [Interviewee 5] |
|  | Subtheme 5.2: retrievability of content independent of time and place | - Quote 35: “You can do it at the times when you just have the opportunity. You don’t have to make a fixed appointment. That’s also then...accommodates this overall situation very well. So you can do it when you’re ready for it.” [Interviewee 5] - Quote 36: “You might not be able to use that at the moment when you’re doing the one element now. You might not need it until three or four months later, and then it comes back to me that I saw it once and that it can be helpful to me at this moment. And then you also have the opportunity to look at the course again if you want to know the details again.” [Interviewee 5] - Quote 37: “So depending on how I would feel if I were to become uncertain about my illness again. It could well be that I would listen again to various topics, for example, with the pain or also the diet again stated and so, because I just now also by the chemo tablets that I now get, I am not allowed to eat various things. I would then probably listen to that again.” [Interviewee 1] |
|  | Theme 6: user-friendliness | - Quote 38: “Yeah, definitely. So I mean I’m a complete layman and so I managed to get through there without much trouble I would already say is user friendly. [...] I found the handling very good. It was actually simple, everything was explained. Well. And actually you could. Actually, you couldn’t do anything wrong.” [Interviewee 1] - Quote 39: “Well, I didn’t find it particularly user-friendly, a bit confusing [...].” [Interviewee 3] |
|  | Subtheme 6.1: customization of the modules | - Quote 40: “I find it less helpful that before you have to do module twelve, you want to have done module three and four and five first because you can’t get there otherwise.” [Interviewee 6] |
|  | Subtheme 6.2: software interface | - Quote 41: “I just thought the layout was nicely done. It’s also a kind of visualization that you walk along a path like that. And it accompanied me on my way through chemo.” [Interviewee 2] - Quote 42: “Very simple. [...] So playful...a little bit in the childish direction. But I think it’s cute. I found that quite nice, because the content is the important thing. The exterior, that of course you can also make much more factual. But I think it’s also a nice distraction. Because this little doll that runs along these paths and somewhere that’s also a path that you walk.” [Interviewee 4] |
|  | Subtheme 6.3: email reminder to increase adherence | - Quote 43: “But up to the point where I, where I no longer want to hear anything, see anything and it was yet, where the message came, the reminder email was yet so man (Person name), come now, do not let you hang. Now you go on.” [Interviewee 1] |
|  | Subtheme 6.4: technical aspects | —^a^ |
|  | Theme 7: recommendations to design the Make It Training intervention to be more appealing | - Quote 44: “But the one with the one with the relatives. Well, that was. That was. If the relatives would pull together, it would be helpful. But when you’re standing there all alone like I am now, well, you do feel upset.” [Interviewee 1] - Quote 45: “And the module for relatives in particular didn’t really help me that much.” [Interviewee 2] - Quote 46: “there are certainly also many patients who are in the same situation as I am, who you could take a bit of a hand psychologically, but mind you, that would have to be tailored to the individual a bit, whether they get that or get the modules or not.” [Interviewee 1] - Quote 47: “Yeah, it was kind of a bit confusing, I just found. Over and over again.” [Interviewee 2] - Quote 48: “I didn’t even know anymore, where was I now? Where had I stopped?” [Interviewee 3] - Quote 49: “It was a couple of things where you had to select something where I didn’t find directly where, where you have to click there to make the check mark come on. So there it was hard to find the spot or I wasn’t on target or.” [Interviewee 1] |

^a^
